# Supplementary material for: Doping dependent intrinsic magnetization in silicon in Ni/Si heterostructures
Source: Sci Rep. 2024 Jan 15;14:1329. doi: 10.1038/s41598-023-50795-w (PMC10789780; doi:10.1038/s41598-023-50795-w)
Supplement: Supplementary file 1 — Supplementary Information. [file 41598_2023_50795_MOESM1_ESM.pdf]

Section I. Resonant longitudinal MOKE hysteresis measurements

Resonant magnetic measurements were performed at the MagneDyn [1] endstation at the externally seeded FERMI Free Electron Laser [2] at Trieste. Analogously to the experimental conditions illustrated in the main article, the samples were placed at an incident angle of 45°, whereas the external saturating magnetic field was along the same direction of the incoming radiation. The reflected XUV pulses, which were resonantly tuned at the Ni $M_{2,3}$ edge at 66.7 eV and at the Si $L_{2,3}$ edge at 99.9 eV, were then collected by a Wollaston-like EUV polarimeter [3]. The collected data allow to reconstruct the resonant magnetization hysteresis at both the edges. As reported in the main article, the signs of the two hysteresis are reversed in sign at the Si $L_{2,3}$ edge. It must be stressed that the intensity of the resonant MOKE hysteresis is sensitive to the exact optical properties of the sample and slight changes of the growing conditions can slightly modify the measured amplitude. A more in-depth discussion of the experimental configuration can be found in [4].
Fig. S1 displays the measured Kerr rotation hysteresis with the probe resonantly tuned at the Ni $M_{2,3}$ (left panel) edge and at the Si $L_{2,3}$ (right panel) for sample HD (red) and LD (blue). A line for the eye has been added on the signal at the Si edge to highlight the hysteresis shape.

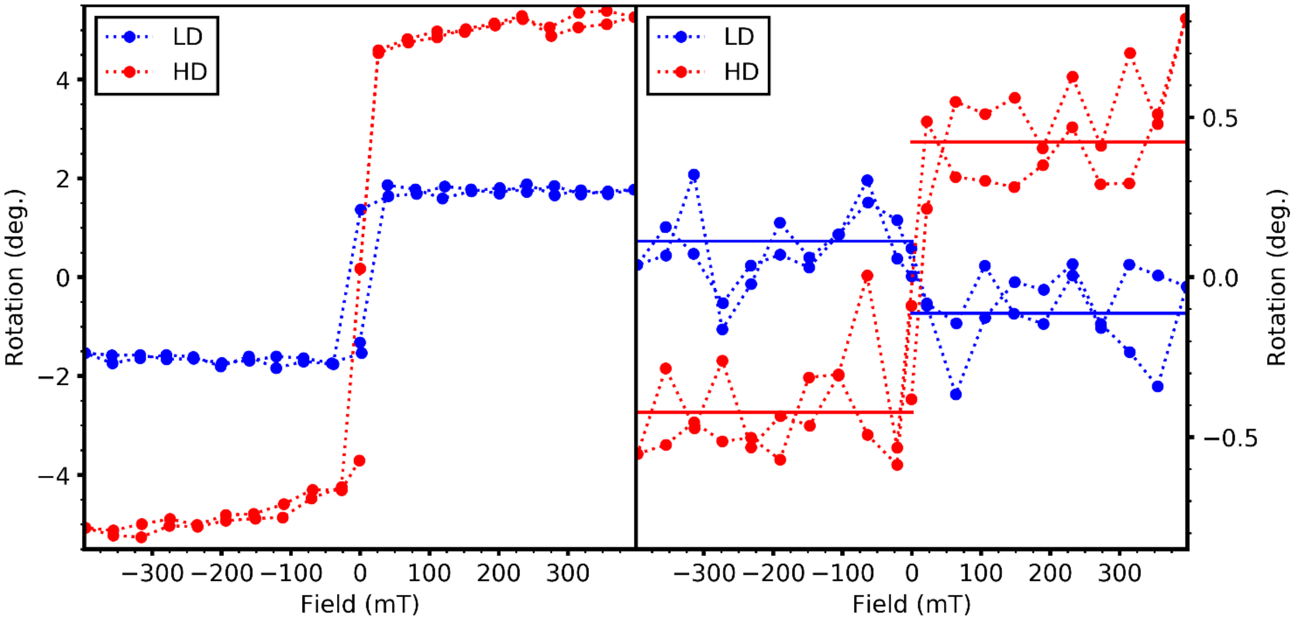

Fig. S1: Kerr rotation for HD and LD samples (red and blue, respectively) at the Ni $M_{2,3}$ edge (left panel) and at the Si $L_{2,3}$ edge (right panel).

Section II: attenuation lengths for Si and Ni.

TEY and Reflectivity spectroscopies are substantially equivalent except for their bulk sensitivity. The figure plots the attenuation length trends for Si and Ni as a function of photon energy. The attenuation lengths at the absorption edges of Ni (red line @66 eV) and Si (blue line @ 100 eV) are significantly different, leading to differing bulk sensitivities in reflectivity at these particular wavelengths. In contrast, when compared with the penetration length of total electron yield, which is only a few nanometers, it becomes evident that it is much more sensitive to the surface than to the bulk. Since the proximity effect manifests in the accumulation zone of silicon near the Si3N4 interface, it is apparent that reflectivity at the Si edge is the more suitable probe for investigating the magnetic state in that specific region of the heterostructure studied in this article.

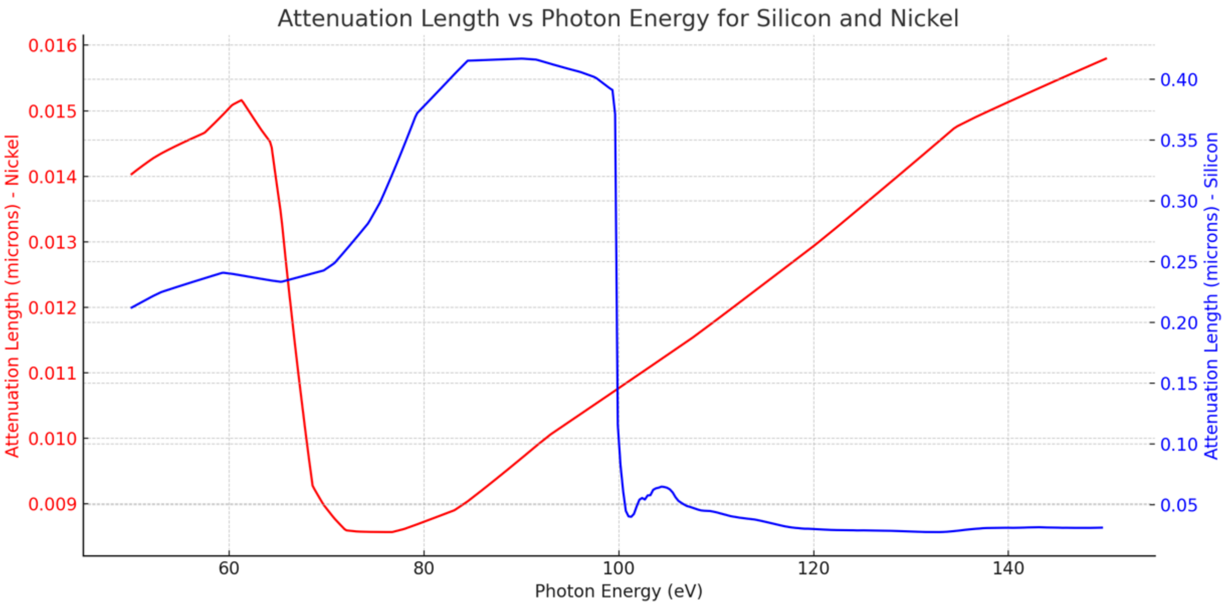

Section III: tunnelling probability across the Si3N4 interlayer

It is possible to estimate the tunnelling probability through a unidimensional potential barrier $V(x)$ within the WKB formalism as

$$T(E_x) = e^{-\theta(E_x)} \ , \ \ \theta(E_x) = \frac{2}{\hbar}\int_{x_1}^{x_2} \sqrt{2m^*(V(x') - E_x)}\, dx'$$

Where the integral shape factor $\theta(E_x)$ is calculated for a particle of reduced mass m^* and longitudinal (to the dimension x) energy E_x within the extrema x_1 and x_2 that are the roots $V(x_1) = V(x_2) = E_x$. This form reflects the lack of an analytic form for the transmission probability for which a general electron barrier profile of the form [5]

$$V(x) = V_0 - Fx + \gamma x^2 - \frac{Q_s}{x}$$

Is adopted, where the linear term refers to an applied potential, the quadratic term accounts to nonlinear corrections and the last term is linked to the effect of the buildup of the image charge Q_s. If we strictly limit to consider the tunnelling through the insulator layer only so that the band bending (typical of the Schottky barrier) can be neglected, the quadratic term can be set to zero. As a result, the tunnelling through the sole insulating layer can be modelled in the approximation of a metal-insulator-metal (MIM) interface. In this case, the constant and the linear term can be replaced by the term $W_M - \chi_I$ and the term $\Phi_B x/L$ respectively, with the justification of the presence of the barrier height Φ_B, whereas W_M and χ_I are respectively the metal work function and the insulator electron affinity. In the same MIM approximation, the image force has to account of an infinite number of image charges and can be represented by the approximated form [5]

$$-\frac{Q_s}{x} \approx -\frac{e^2}{8\pi\varepsilon_0\varepsilon_r}\frac{2}{L}\left[\frac{(x/L)^2}{1-(x/L)^2} + \frac{1 + 2\cdot 0.202\,(x/L)^2 + 2\cdot 0.0372\,(x/L)^5}{2(x/L)}\right]$$

Where ε_r and L are respectively the relative dielectric function and the thickness of the insulating layer, which in the present case is 0.7 nm. Finally, although the linear term of the barrier should be set to be $W_M - \chi_I$, the term has been replaced to the value $E_g/2$, under the assumption that as for

such small thicknesses the insulating layer is not building up any charge it will place its valence and conduction energy bands equally spaced from the metal Fermi energy.

The parameters for the simulation can be found in the next table:

E_g(eV)	Φ_B(eV)	ε_r	m^*/m_0
5.25 [6]	0.02 [7]	6.0 [8]	1.41 [6]

Lastly, to provide a more reliable value of the tunnelling probability, the tunnelling factor $T(E_x)$ has been weighted according to a Fermi-Dirac thermal distribution at 300 K; as the value of Φ_B is above 0, the lower integration extremum for the weighting function has been taken equal to Φ_B . This has allowed to calculate the weighted tunnelling probability as 6.57e-6. However, due to the uncertainty on some of the parameters of the calculation, the probability has been also estimated by also varying the simulation parameters in a range 0.9-1.1 around the mean value, resulting in a total modification of more or less one order of magnitude on the measured value.

[1] M. Malvestuto, A. Caretta, R. Bhardwaj, S. Laterza, F. Parmigiani, A. Gessini, M. Zamolo, F. Galassi, R. Sergo, G. Cautero, M. B. Danailov, A. Demidovic, P. Sigalotti, M. Lonza, R. Borghes, A. Contillo, A. Simoncig, M. Manfredda, L. Raimondi, and M. Zangrando, "The MagneDyn beamline at the FERMI free electron laser", Review of Scientific Instruments 93, 115109 (2022), https://doi.org/10.1063/5.0105261.

[2] E. Allaria, D. Castronovo, P. Cinquegrana, P. Craievich, M. D. Forno, M. B. Danailov, G. D'Auria, A. Demidovich, G. D. Ninno, S. D. Mitri, B. Diviacco, W. M. Fawley, M. Ferianis, E. Ferrari, L. Froehlich, G. Gaio, D. Gauthier, L. Giannessi, R. Ivanov, B. Mahieu, N. Mahne, I. Nikolov, F. Parmigiani, G. Penco, L. Raimondi, C. Scafuri, C. Serpico, P. Sigalotti, S. Spampinati, C. Spezzani, M. Svandrlik, C. Svetina, M. Trovo, M. Veronese, D. Zangrando, and M. Zangrando, "Two-stage seeded soft-X-ray free-electron laser", Nature Photonics 7, 913 (2013), https://doi.org/10.1038/nphoton.2013.277.

[3] A. Caretta, S. Laterza, V. Bonanni, R. Sergo, C. Dri, G. Cautero, F. Galassi, M. Zamolo, A. Simoncig, M. Zangrando, A. Gessini, S. D. Zilio, R. Flammini, P. Moras, A. Demidovich, M. Danailov, F. Parmigiani, and M. Malvestuto, "A novel free-electron laser single-pulse Wollaston polarimeter for magneto-dynamical studies", Structural Dynamics 8, 034304 (2021), https://doi.org/10.1063/4.0000104.

[4] S. Laterza, A. Caretta, R. Bhardwaj, R. Flammini, P. Moras, M. Jugovac, P. Rajak, M. Islam, R. Ciancio, V. Bonanni, B. Casarin, A. Simoncig, M. Zangrando, P. R. Ribic, G. Penco, G. De Ninno, L. Giannessi, A. Demidovich, M. Danailov, F. Parmigiani and M. Malvestuto, "All-optical spin injection in silicon investigated by element specific time-resolved Kerr effect", Optica 9, 12 (2022), https://doi.org/10.1364/OPTICA.471951.

[5] K. L. Jensen, A. Shabaev, S. G. Lambrakos, D. Finkenstadt, N. A. Moody, A. J. Neukirch, S. Tretiak, D. A. Shiffler, and J. J. Petillo, "Analytic model of electron transport through and over non-linear barriers", Journal of Applied Physics 127, 235301 (2020), https://doi.org/10.1063/5.0009759.

[6] Y.-N. Xu and W. Y. Ching, “Electronic structure and optical properties of a and P phases of silicon nitride, silicon oxynitride, and with comparison to silicon dioxide”, Physical Review B 51, 17379 (1995), https://doi.org/10.1103/PhysRevB.51.17379.

[7] M. A. Sobolewski and C. R. Helms, “Properties of ultrathin thermal nitrides in silicon Schottky barrier structures”, Applied Physics Letters 54, 638 (1989), https://doi.org/10.1063/1.100903.

[8] T. A. Pham,T. Li, S. Shankar, F. Gygi, and G. Galli, “Microscopic modeling of the dielectric properties of silicon nitride”, Physical Review B 84, 045308 (2011), https://doi.org/10.1103/PhysRevB.84.045308.
